# Process development options for electronic waste fractionation to achieve maximum material value recovery

**DOI:** 10.1177/0734242X20987895

**Published:** 2021-02-15

**Authors:** Johannes-Robert Bruch, Katrin Bokelmann, Sue M Grimes

**Affiliations:** 1 Department of Civil and Environmental Engineering, Imperial College, UK; 2 Fraunhofer Research Institution for Materials Recycling and Resource Strategies IWKS, Germany

**Keywords:** WEEE, waste fractionation technologies, multi-criteria decision analysis, material recovery from WEEE, critical metals

## Abstract

Revised legislation and bans on imports of waste electrical and electronic equipment (WEEE) into many Asian countries for treatment are driving the need for more efficient WEEE fractionation in Europe by expanding the capacity of treatment plants and improving the percentage recovery of materials of economic value. Data from a key stakeholder survey and consultation are combined with the results of a detailed literature survey to provide weighted matrix input into multi-criteria decision analysis calculations to carry out the following tasks: (a) assess the relative importance of 12 process options against the 6 industry-derived in-process economic potential criteria, that is, increase in product quality, increase in recycling rate, increase in process capacity, decrease in labour costs, decrease in energy costs and decrease in disposal costs; and (b) rank 25 key technologies that have been selected as being the most likely to benefit the efficient sorting of WEEE. The results indicate that the first stage in the development of any total system to achieve maximum economic recovery of materials from WEEE has to be the selection and application of appropriate fractionation process technologies to concentrate valuable components such as critical metals into the smallest possible fractions to achieve their recovery while minimising the disposal costs of low-value products. The stakeholder-based study has determined the priority for viable technical process developments for efficient WEEE fractionation and highlighted the economic and technical improvements that have to be made in the treatment of WEEE.

## Introduction

Waste electrical and electronic equipment (WEEE) is a large and increasingly diverse type of waste with an estimated global yield of approximately 45 million tonnes per annum (Baldé et al., 2017). Particularly in the form of electronic waste (e-waste), it represents a resource-rich stream of increasingly valuable materials that must be carefully managed for the benefit of society as a whole, the environment and public health. It is an extremely difficult waste to handle because it contains a heterogeneous mix of plastics, metals, composites and some hazardous substances that could pose significant risks to human health and the environment (Tsydenova and Bengtsson, 2011). The development of an effective recycling methodology for WEEE has to take into account (a) the requirement to separate the waste into fractions such as plastics, metals and other components that have different economic values in terms of their recyclable content, (b) the fact that the composition of WEEE has changed over the years because of manufacturing and technical changes, including an increase in the amount and number of different plastics used, a reduction in the precious metal content of electronic components, the increasing trend towards miniaturisation and the increased use of embedded batteries, all of which present challenges for recovery and recycling (Tansel, 2017) and (c) any current and future relevant waste legislation. The development of facilities for recovering value from WEEE in the European Economic Area (EEA) would, for example, have to comply with current legislation and governance that addresses three key issues: (a) the definition, collection and treatment of WEEE as set out in the WEEE Directive 2012/19/EU (European Commission, 2012), which defines a common system for the categorisation of electrical and electronic equipment for reporting purposes within the EEA and sets out minimum goals for recycling rates to be used as drivers for governmental efforts to increase collection rates and to incentivise recycling and preparation for re-use; (b) restrictions on transboundary movements of WEEE and materials derived from it within the EEA (European Commission, 2006, 2014a) and to other countries, particularly developing countries (UNEP and Basel Convention, 2014); and (c) restrictions on the treatment of WEEE and of materials derived from it so that end-of-waste criteria in terms of waste and non-waste by-products classifications are met (European Commission, 2008, 2017a). The economics of recovery of value from WEEE in the EEA (Magalini and Huisman, 2018) have been affected by recent legislative changes including (a) the 2018 open scope expansion of the 2012 Directive (European Commission, 2012) that could endanger the achievement of the minimum goals for recycling and (b) the ban on import of waste plastics by Asian countries including China, India, Malaysia and Vietnam that will remove a major market outlet for all recovered plastics including those from WEEE (Malaysian National News Agency Bernama, 2018; Messenger, 2018; Minh, 2018; PRC Ministry of Ecology and Environment, 2017; Press Information Bureau Delhi, 2019; Vietnam Environment Administration, 2018). These definition changes and the restriction of export markets for recovered plastics should, however, incentivise new investment into the recovery of value from WEEE within the EEA.

Most of the recycle value of WEEE, particularly e-waste, lies in the ability to recover the metal content and high-value plastics that are free from any hazardous material contamination. The metal content of e-waste includes base metals, precious metals and economically important critical metals that have risks associated with their supply chains (Deloitte Sustainability et al., 2017; Graedel et al., 2012), including economic availability, political influences, ease of recycling, potential for substitution and likely development of new raw material sources. There are 12 critical metals or groups of metals listed in the 2014 *Report on Critical Raw Materials for the EU* (European Commission, 2014b): antimony, beryllium, chromium, cobalt, gallium, germanium, indium, magnesium, niobium, platinum group metals, rare earth elements, silicon and tungsten. This list contains those of most relevance to WEEE, and although the 2017 listing (European Commission, 2017b) adds further metals to the list, these are not generally found in WEEE. Many publications report on the supply and demand situation with regard to critical metals (Nguyen et al., 2018; Zhang et al., 2017) and on a variety of potential methods for their recovery and recycling from difficult secondary sources (Iȿildar et al., 2018, 2019; Marra et al., 2018, Schaeffer et al., 2016, 2017b; Sun et al., 2017). The need to concentrate valuable materials in WEEE in the smallest possible fractions to maximise recovery potential has now become an important issue. For example, it is necessary to ensure that fractionation makes it possible to remove the hazardous flame-retardant components from plastics (Lateef et al., 2008), and that separation processes used to concentrate specific components such as critical metals in very small fractions take into account techniques that can be used in prioritising their recovery value (Grimes and Maguire, 2020). For reasons such as these, efficient separation of all components containing metal from the plastic fractions, including fibre-reinforced plastics, coated plastics and plastic–metal composites has to be a crucial stage of any WEEE recovery process. The technologies that would be required would have to meet the increasing need for efficient optimal fractionation of WEEE, particularly e-waste, within the constraints of the WEEE Directive to maximise material recovery and value. In the absence of the achievement of optimal fractionation, any cost analysis for material recovery from WEEE would be meaningless. Appropriate technologies for the fractionation of WEEE can be described under four headings: comminution technologies; direct sorting technologies; sensor-based sorting technologies; and hydrometallurgical and chemical methods.

### Comminution technologies

Size reduction of WEEE is normally carried out using mechanical comminution technologies that focus on the reduction of both the mean particle size and the particle size distribution. In state-of-the-art mechanical processing plants, either shredders or crushers without shear or cutting tools (Höggerl, 2015), including those that use rotor chain crushing mechanisms (Linnenkoper and Reintjes, 2017), are used in initial WEEE disassembly operations to facilitate the safe removal of hazardous materials (Salhofer et al., 2016). The second step in the comminution process is usually designed to liberate recyclable material fractions from the WEEE and to adjust the particle size distribution. In many WEEE sorting facilities, hammer or impact mills are used for this purpose (Li et al., 2007a) but novel fractionation technologies such as shock wave electrohydraulic fragmentation (EHF) and electrodynamic fragmentation have also been reported (Bokelmann et al., 2017; Pestalozzi et al., 2018). Such technologies have been shown to achieve a high degree of separation of recyclable fractions from composite material components such as galvanised plastics, printed circuit boards (PCBs) (Bokelmann et al., 2017) and photovoltaic systems (Nevala et al., 2019; Pestalozzi et al., 2018).

### Direct sorting technologies

Separation of WEEE into fractions of known particle size distributions and material composition is a necessary pre-requisite for most automated sorting processes, and there have been a number of reviews that describe the use of both direct sorting technologies and sensor technologies for this purpose (Dalrymple et al., 2007; Wang and Xu, 2014; Zhang and Xu, 2016). Direct sorting technologies for the treatment of WEEE include sieving, use of hydrocyclones (particularly for relatively small particles), air classification, flotation, jigging, electrostatic sorting, magnetic separation and eddy current separation.

Direct sorting applications for the separation of plastic components from WEEE have involved (a) triboelectrostatic separation (Wu et al., 2013) under appropriate conditions for frictional charging, which can lead to the separation of acrylonitrile butadiene styrene (ABS) (Li et al., 2015), low-density polyethylene (LDPE), high-density polyethylene (HDPE), polyethylene terephthalate (PET), polyvinyl chloride (PVC) and polypropylene (PP) from mixed plastics (Silveira et al., 2018), (b) air tables, air classifiers and cyclones for the separation of plastics from WEEE (Pascoe, 2006; Peeters et al., 2014) as part of a copper recovery process, for example (Havlik et al., 2014), (c) air sifting to separate plastics with a high chlorine content from WEEE and end-of-life vehicles (Yoshida et al., 2010), (d) sink–float separation in dense liquid media for sorting plastics (Gent et al., 2011; Menad et al., 2013, Peeters et al., 2014) and (e) froth flotation, with or without chemical treatment of polymer surfaces (Truc and Lee, 2017a, 2017b; Wang et al., 2015a).

Several sorting technologies have been applied to the removal of metals from WEEE (Cui and Forssberg 2003, Veit et al., 2005, 2006). These include magnetic separation to remove ferromagnetic materials, eddy current separation to remove non-ferrous metals including aluminium (Menad et al., 2013; Ruan et al., 2017) and electrostatic corona discharge separation for metal removal (Li et al., 2007b; Zhang and Xu 2016).Various techniques for dense material separation have also been used to improve the selectivity of fractions containing metal from WEEE. These include froth flotation (Estrada-Ruiz et al., 2016), vibration techniques such as pneumatic and hydraulic jigging (Habib et al., 2013; Wang et al., 2015b) and the use of cyclones to improve the separation of metals from shredded PCBs and cable scrap by removing lighter materials such as plastics and fluff (Wang et al., 2015a).

### Sensor-based sorting technologies

A number of remote sensing technologies that are sufficiently fast in detecting and removing target particles have been validated for the sorting of WEEE. When these technologies are used, high levels of differentiation that can enhance the separation and value of recovered material can be achieved (Dalrymple et al., 2007). Sensor-based technologies include the following: the use of colour cameras and 3D-scanners for sorting specific components of WEEE based on image recognition (Arends et al., 2015; Picón et al., 2012); the use of colour cameras for sorting non-ferromagnetic metals (Kutila et al., 2005) and plastics (Buekens and Yang 2014), infrared (IR) (Beigbeder et al., 2013); Fourier Transform IR spectroscopy (FTIR) (Taurino et al., 2010), combined IR and colour sensing (Menad et al., 2013, Tange et al., 2013) for the selective separation of different types of plastics; ultraviolet–visible (UV–Vis) spectroscopy for separating out glass fractions that are commonly used in cathode ray tubes (Huber and Pansinger 2011); hyper-spectral imaging in the visible and near-infrared range for separating non-ferromagnetic metals from shredded WEEE (Candiani et al., 2017); Raman spectroscopy for the detection of brominated flame retardants (BFRs) in thermoplastics (Taurino et al., 2010); X-ray fluorescence and X-ray transmission spectroscopy for detecting WEEE fractions that contain heavy elements including metals (Braibant et al., 2018; Buzanich, 2016; Taurino et al., 2010) and BFRs (Taurino et al., 2010); electromagnetic induction sensors for detecting and separating out metals from shredded television scrap; and laser-induced breakdown spectroscopy for classifying plastics and critical metals in WEEE (Costa et al., 2017; Noll et al., 2018; Schlummer et al., 2016).

### Hydrometallurgical and chemical methods

Hydrometallurgical methods have been used in the recovery of metals from WEEE (Awasthi et al., 2017; Dalrymple et al., 2007; Havlik et al., 2011; Zhang and Xu, 2016) but have been subject to criticism on environmental grounds because of their extensive use of extractants, solvent stripping agents, etc. (Hadi et al., 2015, Iannicelli-Zubiani et al., 2017). Chemical methods, however, have been used in successful high-value metal recovery processes (Schaeffer et al., 2017a, 2017b), and to recover high-quality polymers through the removal of BFRs by using ionic liquids (Lateef et al., 2008) and other solvent processes (Schlummer et al., 2016). The successful use of processes based on chemicals relies particularly on the efficient separation of the fractions of WEEE that contain metals to produce feedstocks that can be treated for metal recovery with the minimum use of process chemicals.

With increasing tonnages of WEEE requiring treatment, and recent import bans on wastes by Asian and Far Eastern countries, there is a need within the EU to develop process technologies for efficient WEEE fractionation and separation that take into account technical and legislative requirements in the EU, including the safe removal of any hazardous materials. We now report on the analysis of stakeholder-based survey data combined with a detailed literature survey to prioritise viable WEEE process development options and rank technologies that concentrate valuable materials in the smallest possible fractions to permit maximum economic recovery and separation of commercially viable materials.

## Methodology

The methodology used to obtain stakeholder data to enable the evaluation of viable WEEE process development options, particularly for e-waste, consisted of four parts: (a) a detailed literature analysis of both technologies that have already been developed and those under development that can be applied to separating the components of WEEE; (b) selection of key stakeholder participants; (c) design of a questionnaire to collect stakeholder data on process development options for WEEE; and (d) data handling and analysis.

### Literature analysis

A detailed review of the primary and secondary literature and technical documents using critical citation keyword searches was carried out to determine what relevant fractionation technologies are available to the WEEE treatment industries. The aim of the review was to identify technologies that were likely to achieve the efficient fractionation of WEEE, particularly e-waste, within the constraints of the WEEE Directive to maximise material recovery and value. The technologies and methods identified were classified into four main groups: comminution; direct sorting; sensor-based sorting; and hydrometallurgical and chemical methods. The results from the literature review were analysed in detail to produce a subset of 25 current and emerging technologies identified as being of particular relevance to the separation and fractionation of WEEE in terms of achieving selective recovery of economically viable materials. Consultation with the 33 key stakeholders led to a classification of these technologies in terms of the key process options (chosen for their function) likely to effect the successful separation and fractionation of high-value materials from WEEE: metals, engineered plastics, glass and composite materials of value. A set of key process options was then identified through a combination of the literature survey and detailed discussions with relevant industry stakeholders: removal of specific molecules; separation of component materials of composites; comminution; sizing; removal of ferromagnetic metals; removal of non-ferromagnetic metals; removal of plastics; removal of glass and ceramics; sorting ferromagnetic metals; sorting non-ferromagnetic metals; sorting plastics; and sorting glass and ceramics.

### Selection of key stakeholder participants

The 33 stakeholders were selected from the network of the authors’ contacts within the European WEEE management sector, and all of them held senior management positions (as directors or operations managers) with responsibility for managing automated WEEE sorting processes in Europe within their organisations. All the stakeholders were users of relevant technologies for the separation of target materials from WEEE, had a sound understanding of the relevant economic and technical aspects of such processes and had responsibility for key decisions with regard to investment in the company’s technology portfolio.

Engagement with the stakeholders included discussions on (a) the findings from the literature survey to assess their opinion on the selection of the technologies most likely to be successful in separation of materials, (b) the relevant key process options for WEEE fractionation and (c) the economic process criteria that should be used in the data analysis. Each stakeholder was invited to participate in the survey, which was designed to determine the practitioner’s point of view with regard to the relative importance of technologies available to the WEEE treatment industries. Direct input from the 33 stakeholders obtained in this part of the research resulted in endorsement of the concept of the research, the selection of technologies and process options to be used and the measures against which the options should be judged. The 33 stakeholders also decided on what cost priorities they regarded as important in the technology survey, expressed in Table 2 as economic potential criteria in the weighted analysis set used to prioritise technologies for the separation of WEEE fractions.

### Survey questionnaire

A questionnaire was designed to obtain meaningful and unbiased data from the key stakeholders who had responsibility for managing automated WEEE sorting processes in Europe. To eliminate bias, the stakeholders selected for inclusion in the survey represented the different activities and priorities that exist across the electrical and electronics recycling industries, for example, companies that focus on specialist processes such as plastics recovery or sorting of non-ferrous metals, and those that treat and recycle all fractions and components of WEEE. The questionnaire consisted of two parts and it was supplied to relevant stakeholders in German or English, as appropriate, for completion either by means of an interview or email response, and the survey was conducted over a period of four months.

Of the 33 stakeholders, 21 confirmed their agreement to participate in the survey, and the surveys were sent by email (personalised) to each stakeholder for completion either by email or via a telephone or face-to-face interview. Of the 21 stakeholders, 10 responded in full, matching their weightings against the agreed criteria. The remaining 11 stakeholders provided partial responses that added to the information provided in the full responses.

The first part of the questionnaire was designed to gain an understanding of the nature of the activities of the stakeholder and an insight into the WEEE collection streams and the separated fractions of secondary raw materials derived from WEEE by individual stakeholder organisations. The following set of open-ended questions was used:

1. What collection streams of WEEE do you accept?
2. What module/component fractions of WEEE do you accept?
3. What material fractions of WEEE do you accept?
4. Which of the material streams that you produce incur disposal costs?
5. Which of the material streams that you produce generate revenue?
6. In your answers to questions 1–5, which of the materials (modules, components, fractions) mentioned do you expect to have relatively high potential for economic improvement?

In the second part of the questionnaire, stakeholders were required to complete two data input sheets providing relative numerical scores between 1 and 10 for (a) the 12 key process options, chosen to represent the methods used in the separation of WEEE fractions to optimise total value recovery, matched against a set of 6 criteria (defined as ‘industry-derived in-process material costs’) that describe economic potential (Table 1), and (b) the importance they attached to each of the 6 criteria (Table 2). The numerical data supplied in the questionnaire were also elaborated and endorsed by stakeholders via a comment box designed to provide additional feedback. The weighted stakeholder data provide input into the calculations that can be analysed mathematically to aid management decisions on the prioritised options for future development.

**Table 1.** Stakeholder input data – weighted priorities on a scale from 1=low to 10=high against the six criteria, with data from a specific stakeholder inserted as an example.

Key process options	Economic potential criteria					
	Increase in product quality	Increase in recycling rate	Increase in process capacity	Decrease in labour costs	Decrease in energy costs	Decrease in disposal costs
Removal of specific modules	5	1	1	1	1	5
Separation of component materials of composites	3	3	1	1	1	3
Comminution	1	1	1	1	1	1
Sizing	1	1	1	1	1	1
Removal of ferromagnetic metal	1	1	1	1	1	1
Removal of non-ferromagnetic metal	1	1	1	1	1	1
Removal of plastics	5	1	1	1	1	3
Removal of glass and ceramics	5	1	5	1	5	1
Sorting ferromagnetic metal	1	1	1	1	1	1
Sorting non-ferromagnetic metal	1	1	1	1	1	1
Sorting plastics	7	1	1	1	1	3
Sorting glass and ceramics	1	1	1	1	1	1

**Table 2.** Stakeholder input data – weighted priorities on a scale from 1=low to 10=high of criteria used in assessment of process development options with data from the same stakeholder as Table 1 inserted as an example.

Relative importance of each criterion	Economic potential criteria					
	Increase in product quality	Increase in recycling rate	Increase in process capacity	Decrease in labour costs	Decrease in energy costs	Decrease in disposal costs
	5	1	1	1	1	5

### Data handling and assessment

Questions 1 to 6 in the first part of the questionnaire are evaluated by ranking the most common keywords (system, component and material descriptors and the words ‘all’ and ‘none’) found in the stakeholder responses on the basis of the number of times they appear. Identified keywords are counted only if they occur more than once throughout all stakeholder responses, and repetition of any keyword by the same stakeholder in answer to the same question is counted only once. Because questionnaires were answered by stakeholders from different European countries, in selecting keywords the differences in definitions of WEEE collection streams in individual national member state countries were taken into account.

The weighted matrix information supplied by individual stakeholders in the second part of the questionnaire provides data that can be analysed mathematically to aid management decisions. Examples of this type of approach in waste management decision-making include use of a weighted matrix analysis to develop a hierarchy of waste management options for the treatment of the organic waste fraction of municipal waste (Donaldson et al., 2001), benchmarking to determine the benefits of environmental recycling within the secondary metal sector, (Grimes et al., 2008, 2016), sensitivity analysis to determine the best strategic waste treatment technology mix in Thailand (Grimes and Tanpoonkiat, 2013), evaluation and selection of treatment strategies for WEEE (Bereketli et al., 2011) and multi-criteria group decision-making for evaluating the performance of e-waste recycling programmes under uncertainty (Wibowo and Deng, 2015).

The numerical data obtained from stakeholders were analysed using multi-criteria decision analysis (MCDA), an analytical approach designed to deal with the difficulties that human decision-makers have in handling large amounts of complex information in a consistent way to identify best options (Coelho et al., 2017; Dodgson et al., 2009; Rezaei, 2015; Valasquez and Hester, 2013). The guidelines for MCDA, particularly on criteria scoring and weighting, have been set out in the comprehensive manual Multi-criteria Analysis (Dodgson et al., 2009). A key assumption made in MCDA is the mutual independence of preferences so that it is possible to assign scores for each option according to one criterion in the absence of knowledge of the scores for that option according to any other criterion. To achieve this, all criteria scores should be assigned independently. In considering the 12 potential process development options for WEEE listed in Table 1, for example, MCDA defines the individual options as $o_{i\in I}$ ( I={1,…,12} ), within a finite set of options

$$O=o_{1},o_{2},\ldots ,o_{i},\ldots ,o_{12}$$

in which each option has to be judged individually against each criterion $p_{j\in J}$ ( J={1,…,12} ) within the set of six economic potential criteria listed in Table 1:

$$P=p_{1},p_{2},\ldots ,p_{j},\ldots ,p_{6}$$

Each criterion is also assigned a weighting $w_{i}$ , characterising its importance relative to the other criteria within the set. The weighted score for each option for a given criterion is the product of the stakeholder input value and the criterion weighting, and the weighted scores can be normalised for any criterion to give a value that can then be used to combine all input data to assign a priority score to the process options with greatest economic potential. The stakeholders were asked to supply weighted data by assigning values from 1 to 10. Any nil responses were assigned a value of 1.

In this work, the MCDA was carried out by normalising the data supplied by stakeholders for both the economic potential criteria and the weightings they attributed to the relative importance of each criterion using equations (1) and (2).

The normalised economic potential is given by

(1) $$n_{i,j,k}=\left\{ \begin{array}{l} 5.5,\;if\;max_{\hat{l}\varepsilon I}p_{\hat{l},j,k}=min_{\hat{l}\varepsilon I}p_{\hat{l},j,k,}\\ 1+9.\;\frac{p_{i,j,k}-min_{\hat{l}\varepsilon I}p_{\hat{l},j,k}}{\;max_{\hat{l}\varepsilon I}p_{\hat{l},j,k}-min_{\hat{l}\varepsilon I}p_{\hat{l},j,k}},\\ \;if\;max_{\hat{l}\varepsilon I}p_{\hat{l},j,k}\neq \;min_{\hat{l}\varepsilon I}p_{\hat{l},j,k,} \end{array}\right.$$

where $p_{i,j,k}$ is the economic potential of the key process option $o_{i}$ in Table 1 with respect to each criterion $p_{j}$ listed in Table 1 from data supplied by stakeholder $s_{k}$ , where $s_{k\in K}$ ( K={1,…,n} ) is an element of set S ( $S=s_{1},s_{2},\ldots ,s_{k},\ldots ,s_{n}$ ) that contains all n stakeholders who completed and returned the questionnaire.

The normalised weightings of the economic potential for the criteria $p_{j}$ for each stakeholder $s_{k}$ are given by

(2) $$w_{j,k}=\left\{ \begin{array}{l} 5.5,\;if\;max_{\hat{J}\varepsilon J}d_{\hat{J}k}=min_{\hat{J}\varepsilon J}d_{\hat{J},k},\\ 1+9.\;\frac{d_{j,k}-min_{\hat{J}\varepsilon J}d_{\hat{J},k}}{\;max_{\hat{J}\varepsilon J}d_{\hat{J},k}-min_{\hat{J}\varepsilon J}d_{\hat{J},k}},\\ \;if\;max_{\hat{J}\varepsilon J}d_{\hat{J},k}\neq \;min_{\hat{J}\varepsilon J}d_{\hat{J},k,} \end{array}\right.$$

where $d_{j,k}$ is the relative importance score assigned to criterion $p_{j}$ by stakeholder $s_{k}$ .

A dimensionless priority ranking score for each key process option $o_{i}$ . and stakeholder $s_{k}$ can then be calculated from the following summation

(3) $$a_{i,k}=\sum_{j\in J}n_{i,j,k}\cdot w_{j,k}.$$

For each option in set O , the median over all stakeholders in set S is calculated. These medians are used to rank the options in set O . The results of this MCDA on the ranking of process options against economic criteria can then be combined with the information obtained from the literature and consultation survey that identifed 25 technologies to rank in order of their likely overall benefit to the separation of components of WEEE. The numerical inputs into this calculation consisted of (a) the dimensionless priority ranking score for each key process option obtained from the MCDA, and (b) a relevance indicator defining the number of process options to which each technology can be applied based on the results of the literature and consultation survey.

The 25 technologies in set T ( T=t _1_ $,...,t_{l},...,t$ _25_) identified as most likely to be successful for use in one or more of the 12 key process options ( $O=o_{1},...,o_{12})$ can be ranked using the results of the MCDA and a relevance indicator $r_{l,i}$ in equation (4)

(4) $$t_{l}=\sum_{i\in I}m_{i}\cdot r_{l,i},$$

where $t_{l}$ is a dimensionless ranking score, $m_{i}$ is the median desirability of the process option $o_{i}$ determined in the MCDA analysis and $r_{l,i}$ is a relevance indicator that is given a value of 1 in cases in which there is reported evidence for the use of the technology in a process option and a value of 0 in cases in which there is no such evidence. The information on whether reported evidence is found for the use of a technology in a process option is summarised in Table 3, and in using equation (4), an assumption is made that priority over information from other potential sources such as the literature should be given to any technologies that are highly rated by stakeholders in the data input.

**Table 3.** The technologies identified as those most likely to benefit the 12 key process options for sorting WEEE.

Identified technologies	Relevant process options
Chain tool comminution	Comminution
Colour camera	Removal of specific modules; sorting non-ferrous metal; sorting plastics
Dry density-based sorting	Removal of plastics; sorting plastics
ECD separation	Removal of non-ferromagnetic metal
Eddy current separation	Removal of non-ferromagnetic metal
EHF	Separation of component materials of composites; comminution
EM induction sensor	Removal of ferromagnetic metal; removal of non-ferromagnetic metal
Ferromagnetic separation	Removal of ferromagnetic metal
Froth flotation	Sorting plastics
FTIR spectroscopy	Sorting plastics
Hydrocyclone	Sorting
Laser scanner	Removal of specific modules
LIBS	Removal of specific modules; Sorting non-ferromagnetic metal; Sorting plastics
NIR spectroscopy	Removal of plastics; Sorting plastics
Raman spectroscopy	Sorting plastics
Shredding and milling	Comminution
Sieving	Sizing
Smasher	Comminution
Terahertz spectroscopy	Sorting plastics
Triboelectrostatic separation	Sorting plastics
UV–Vis spectroscopy	Sorting glass and ceramics
VNIR spectroscopy	Sorting non-ferromagnetic metal
Wet density-based sorting	Sorting non-ferromagnetic metal; sorting plastics
XRF spectroscopy	Sorting non-ferromagnetic metal; sorting plastics
XRT spectroscopy	Sorting non-ferromagnetic metal

WEEE: waste electrical and electronic equipment; ECD: electrostatic corona discharge; EHF: electrohydraulic fragmentation; EM: electromagnetic; FTIR: Fourier transform infrared; LIBS: Laser-induced breakdown spectroscopy; NIR: near-infrared; UV–Vis: ultraviolet–visible; VNIR: Visible and near-infrared; XRF: X-ray fluorescence; XRT: X-ray transmission.

### Sensitivity analysis

The results reported here are those from a study designed to assess which existing and developing technologies are most likely to lead to the efficient fractionation of WEEE, particularly e-waste, into size fractions that would maximise the efficiency of material recovery from these wastes. The results are specific to the situation in the EEA, but the methodology is general and can be applied to other situations with appropriate stakeholder input. In all analyses in which methodologies such as MCDA are used to overcome the difficulties that human decision-makers have in handling large amounts of complex and often uncertain information in a consistent way, sensitivity analyses can be used to show how changes in input data can affect the output (Grimes and Tanpoonkiat, 2013; Grimes et al., 2008). An important feature of the input data into the MCDA used in this study is that they ensure that stakeholder weightings of criteria and rankings are obtained from a wide variety of industry interests with final input data being agreed values. Sensitivity analyses can be carried out by varying the input data by specific percentages above or below agreed values, by varying the importance of stakeholder rankings or by use of specialist groups of stakeholder expertise to account for different input parameters arising from, for example, regional economic and political issues (Grimes and Maguire, 2020). As an example in the current study, if the importance of all stakeholder rankings under the economic potential criterion *labour costs* is arbitrarily reduced by 2, the only change in the top 10 technology rankings from this study is that EHF moves from position 7 to position 5.

## Results and discussion

**Figure 1.**
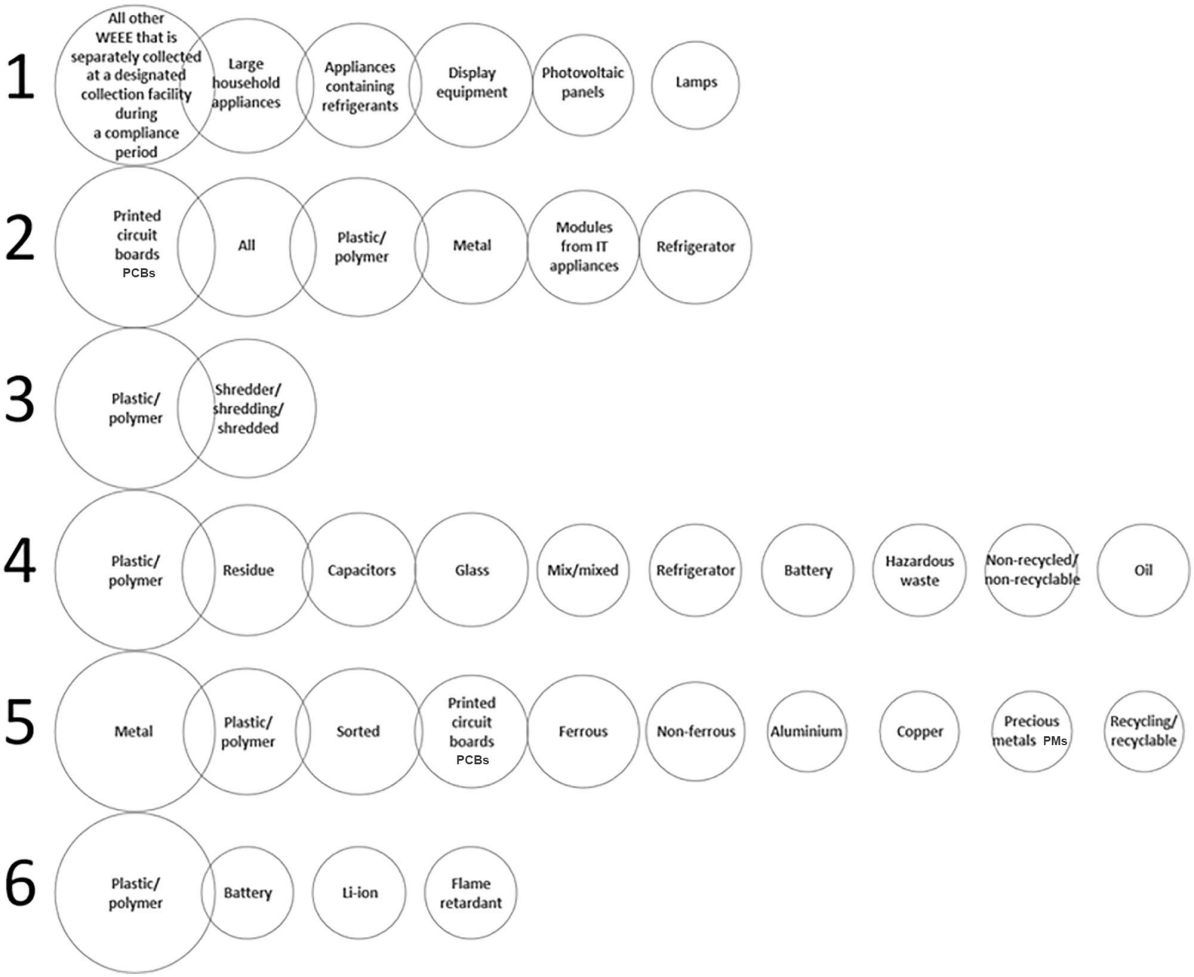
Most common keywords occurring in responses to the questions 1 to 6. Within each question, areas of circles are proportionate to the numbers of stakeholders by which the keywords were mentioned. WEEE: waste electrical and electronic equipment; PCBs: printed circuit boards; PMs: precious metals.

**Figure 2.**
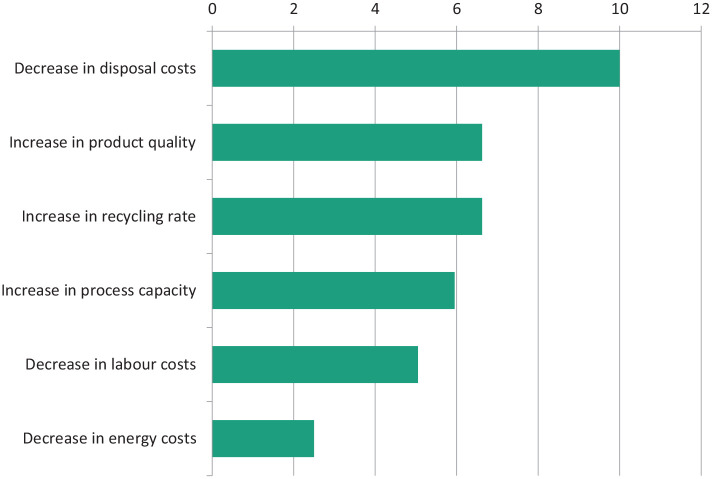
Ranking of the six identified important economic criteria.

Data obtained from a detailed relevant literature survey and key stakeholder consultations have been combined with those from a stakeholder questionnaire and constitute the input into MCDA calculations (Tables 1 and 2) to assess technologies that could achieve optimal fractionation of WEEE with the aim of maximising material recovery for recycling. The literature review and stakeholder consultation survey identified a set of 12 key process options and a set of 25 technologies (Table 3) that were most likely to benefit the key process options required for optimal fractionation of WEEE. Although many of the selected technologies are related, all of them can be used independently from one another. The information collected from the questionnaires on stakeholders’ activities in WEEE separation and treatment is shown in Figure 1 and the results of stakeholder ranking of the relative importance of six identified economic potential criteria (*increase in product quality, increase in recycling rate, increase in process capacity, decrease in labour costs, decrease in energy costs* and *decrease in disposal costs*) are given in Figure 2.

The MCDA calculations involved (a) ranking of the 12 key process options against a set of stakeholder industry-derived process economic criteria, which were chosen for their potential importance in achieving optimum fractionation success, and (b) ranking of the 25 technologies most likely to benefit one or more of the 12 key process options. The numerical input data required in the evaluation of the rankings were provided by stakeholders in the form of weighted matrix information on two parameters, first, as illustrated in Table 1 for a specific stakeholder, recording the relative importance of the 12 key process options against the six economic potential criteria and, second, the relative importance attached to the individual criteria, as illustrated in Table 2 for the same stakeholder.

The data obtained from the stakeholder survey have been combined with the findings from a detailed literature review and stakeholder consultation exercise using the analysis described in equation (4) to prioritise viable WEEE process development options for efficient WEEE fractionation to permit maximum high-quality material recovery (Figure 4). The data in Figure 1 for responses to questions 4–6 of the stakeholder questionnaire show how industry stakeholders rank fractions of WEEE that (a) incur disposal costs, (b) generate revenue and (c) on removal would benefit the overall economics of recovery. It is clear that although efficient separation of plastics and polymers in terms of revenue potential can lead to recovery of high-value engineering plastics such as PP, polystyrene, ABS and blends of ABS with polycarbonates (PC), this is counteracted by the high disposal costs of low-value polymer fractions.

The results of a normalised analysis (Figure 2) of the assessment of the relative importance that stakeholders attach to the economic criteria show that these are ranked as follows: *decrease in disposal costs > increase in product quality = increase recycling rate > increase in process capacity > decrease in labour costs > decrease in energy costs*. Although the criteria may not be totally independent, for example, the three criteria *increase in product quality, increase in recycling rate* and *decrease in disposal costs* may have mutual dependency, it is clear that in any process, the cost of disposal of low-quality materials has a major effect on the economics of efficient recycling of WEEE, leading to *decrease in disposal costs* having the highest ranking. Achieving a reduction in disposal costs is seen as the most desirable of the economic factors affecting recovery of value from WEEE, but there will always be a conflict between the disposal and process costs involved, for example, automated removal of black plastics on an industrial scale, which would reduce the disposal costs of low-grade plastics created as a by-product from separation processes designed to recover high-value engineering plastics, is not currently carried out.

**Figure 3.**
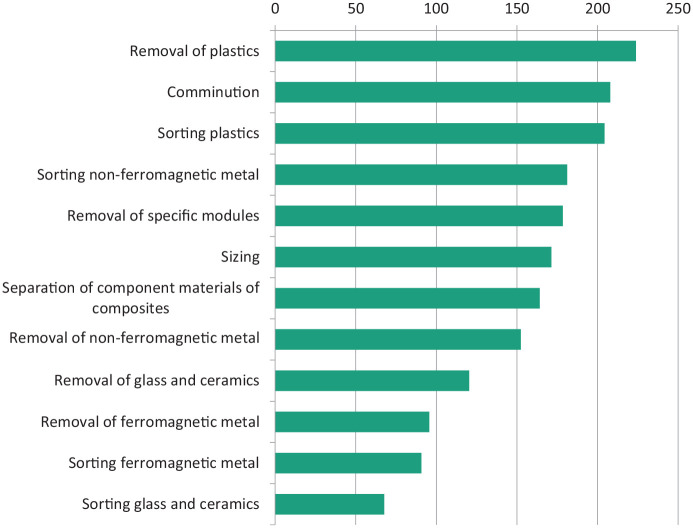
Ranking of the relative importance of the twelve process options.

**Figure 4.**
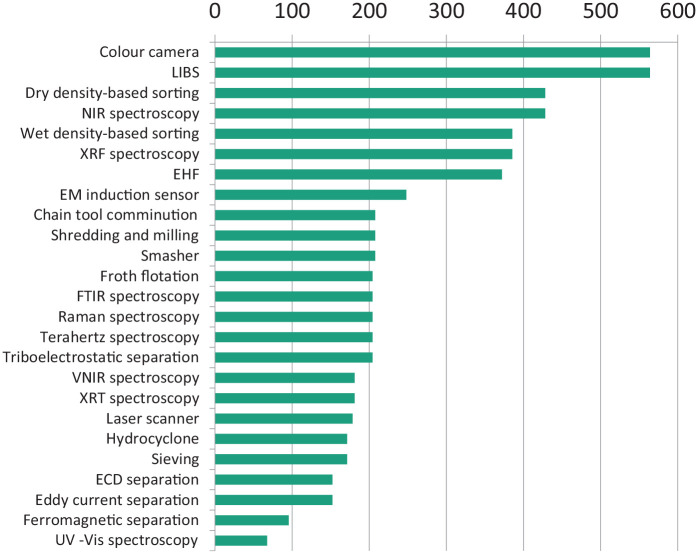
Ranking of twenty-five technologies. LIBS: Laser-induced breakdown spectroscopy; NIR: near-infrared; XRF: X-ray fluorescence; EHF: electrohydraulic fragmentation; EM: electromagnetic; FTIR: Fourier transform infrared; VNIR: visible and near-infrared; XRT: X-ray transmission; ECD: electrostatic corona discharge; UV–Vis: ultraviolet–visible.

The information from stakeholder numerical data input into an MCDA calculation is shown in Figure 3 as a ranking of the relative importance of the 12 key process options identified in the overall WEEE fractionation process for achieving optimum efficiency and economic value, with the removal and sorting of plastics shown to be a key factor in achieving overall efficient recovery of high-quality material. Combining the output from the MCDA with relevance indicators for the 25 technologies identified as those likely to be successful in one or more of the 12 key process options results in the ranking of the technologies as shown in Figure 4. Sensor-based sorting methods, density-based sorting and advanced mechanical dismantling methods such as EHF are ranked as the most versatile technologies with respect to process development and improvement; overall economic efficiency in the recovery of material value from WEEE is most likely to arise from improvements in the use of these technologies. They are particularly relevant for the removal and fractionation of the plastics components of WEEE, but there is also a perceived need to develop automatic technologies to remove and separate other WEEE fractions including the composite type of glass that is part of modern display units. This, like plastics, is not currently recycled, but incurs substantial disposal costs.

Although the results of the study of the technologies most likely to achieve optimal fractionation of WEEE to achieve maximum material recovery for recycling are specific to the European situation, the methodology developed is general, and with appropriate stakeholder input can be applied to any region, country or recovery operation.

## Conclusion

The treatment of large tonnages of the heterogeneous waste streams from end-of-life electrical and electronic goods has become an increasing problem for the recovery and recycling industries. Recovery of value from these wastes, however, is complicated both by their complexity and by national and international legislation applied to the treatment processes used and to any materials derived from these. In Europe, there is now a requirement to expand the capacity of treatment plants and improve the percentage recovery of materials with economic value. The need to concentrate valuable materials in WEEE into the smallest possible fractions to maximise recovery potential is now an important issue; for example, this will ensure that treatment after fractionation makes it possible to remove hazardous flame-retardant components from plastics, and also that specific components such as critical metals are concentrated into very small fractions to achieve maximum recovery potential. For reasons such as these, efficient separation of components containing metal from plastics has to be a crucial stage in any WEEE recovery process.

The initial stage in the development of total systems to achieve maximum economic recovery of materials from WEEE has to be the selection and application of appropriate fractionation process technologies. A combination of stakeholder-based survey data with a detailed literature survey has been used in this study to provide weighted matrix input data into MCDA calculations to prioritise and rank both process options and technologies available that could achieve optimal fractionation of WEEE with the aim of maximising material recovery for recycling. The literature review and stakeholder consultation survey permitted the ranking of 12 key process options and 25 technologies against the following six in-process economic criteria (*increase in product quality, increase in recycling rate, increase in process capacity, decrease in labour costs, decrease in energy costs* and *decrease in disposal costs*), which were selected by the key stakeholders as being the most relevant in-process cost considerations that would lead to the optimal fractionation of WEEE. The results of this normalised analysis of the assessment of the relative importance that stakeholders attach to the economic criteria show that these are ranked as follows: *decrease in disposal costs > increase in product quality = increase recycling rate > increase in process capacity > decrease in labour costs > decrease in energy costs*. Although the criteria may not be totally independent it is clear that in any process, the cost of disposal of low-quality materials will have a major effect on the economics of efficient recycling of WEEE, and that any fractionation processes developed must take account of this. The 25 technologies were also ranked using a combination of the results of the process option rankings and a stakeholder-determined relevance indicator.

This stakeholder-based study has (a) determined the priorities for viable technical process developments for efficient WEEE fractionation for maximum recovery of high-quality material, and (b) highlighted the economic and technical improvements in WEEE treatment that will have to be made at a time when European electronics recyclers will be required to expand their facilities because of increased volumes of WEEE and new import bans on wastes by Asian countries. Although it is important that fractionation processes have the ability to recover metal content (including precious metals and critical metals) from the smallest fractions possible to achieve maximum economic recovery, it is also important as far as the European situation is concerned that the processes are capable of recovering high-quality economically recyclable plastic fractions that are free from hazardous flame-retardant materials.

The results of the study of the technologies most likely to achieve optimal fractionation of WEEE to achieve maximum material recovery for recycling are specific to the European situation, but the methodology developed is general, and with appropriate stakeholder input it can be applied to any region, country or recovery operation.
